# Epidémiologie de l'infection à *Helicobacter Pylori* à Yaoundé: de la particularité à l’énigme Africaine

**DOI:** 10.11604/pamj.2013.16.115.3007

**Published:** 2013-11-25

**Authors:** Firmin Ankouane Andoulo, Dominique Noah Noah, Michèle Tagni-Sartre, Elie Claude Ndjitoyap Ndam, Katleen Ngu Blackett

**Affiliations:** 1 CHU de Yaoundé, Département de Médecine Interne et Spécialités, Faculté de Médecine et des Sciences Biomédicales, Université de Yaoundé I, Cameroon; 2 Hôpital Central de Yaoundé, service de gastroentérologie; Faculté de Médecine et des Sciences Pharmaceutiques, Université de Douala, Cameroon; 3 Centre Médical la Cathédrale, Yaoundé, Cameroon

**Keywords:** Helicobacter pylori, épidémiologie, énigme Africaine, pays en voie de développement, Cameroun, Helicobacter pylori, Epidemiology, African enigma, developing countries, Cameroon

## Abstract

**Introduction:**

L'infection à *Hélicobacter pylori* concerne la moitié de la population mondiale, principalement dans les pays en voie de développement où l'infection atteint 80% de la population. Le but de notre étude était de déterminer la prévalence de l'infection à *Hélicobacter pylori* et de mettre en évidence les déterminants de l'infection ainsi que les maladies associées au Cameroun.

**Méthodes:**

L’étude concernait 171 sujets symptomatiques référés pour une fibroscopie œsogastroduodénale au Centre Hospitalier et Universitaire de Yaoundé et au Centre Médical la Cathédrale. L'infection à *H.pylori* était objectivée par un test rapide à l'uréase kit commercial Pronto Dry^®^ (Medical Instruments Corporation, Solothurn, Switzerland).

**Résultats:**

La prévalence globale de l'infection à *Hélicobacter pylori* était de 72,5% (124/171) (Intervalle de Confiance (IC) à 95% 65,2-79,1%) et dans chaque groupe d’âge (moins de 40ans, 40-50ans, plus de 50ans) de 83,1%(64/77), 67,4%(29/43) et 60,8%(31/51) respectivement. En analyse univariée, le risque de l'infection était significativement élevé dans le groupe de moins de 40ans comparé au groupe de 40-50ans (Risque Relatif (RR) 0,42 IC 95% 0,16-1,1, p = 0,04 pour le groupe 40-50ans) et comparé au groupe de plus de 50ans (RR 0,73; 0,57-0,93, p = 0,004 pour le groupe de plus de 50ans). La prévalence de l'infection à *H.pylori* était de 63,0% (17/27) pour l'ulcère duodénal, 50%(4/8) pour l'ulcère gastrique et 100%(2/2) pour le cancer gastrique.

**Conclusion:**

A prévalence de l'infection à *H.pylori* au Cameroun est très élevée et significativement liée à l’âge de moins de 40 ans.

## Introduction

C'est en 1982 que J. Robin Warren et Barry J. Marshall ont identifié une bactérie qui colonisait la muqueuse gastrique et qu'ils ont appelée *Helicobacter Pylori (H.pylori)* [1]. On estime qu'environ la moitié de la population mondiale est infectée par H.pylori et principalement dans les pays en voie de développement. Dans ces pays, environ 80% de sujets sont infestés dès l'enfance et restent infectés toute la vie [2, 3]. Le problème de l'infection à *H.pylori* réside sur ses associations à certaines maladies gastroduodénales dont l'ulcère, la gastrite chronique, les lymphomes de MALT et le cancer gastrique [4, 5]. D'autres associations entre l'infection à *H.pylori* et certaines maladies extradigestives ont été signalées [6, 7].

Le bas niveau socioéconomique, l'environnement surpeuplé des villes, la jeunesse de la population et la vie en communautés sont des facteurs de risque à l'infection par *H.pylori* [3]. Peu d’études existent au Cameroun traitant du problème de l'infection à H.pylori et de ses conséquences sur la santé de la population [8, 9].

A partir d'une étude prospective menée à Yaoundé auprès des sujets symptomatiques, nous avons voulu déterminer la prévalence de l'infection à *H. Pylori* et mettre en évidence les déterminants de l'infection ainsi que les maladies gastroduodénales associées.

## Méthodes

Etude prospective menée du 29 octobre 2012 au 30 janvier 2013 au Centre Hospitalier et Universitaire (CHU) de Yaoundé et au Centre Médical la Cathédrale (CMC) de Yaoundé dans une population de malades qui étaient référés pour un examen de fibroscopie œsogastroduodénale (FOGD) pour des signes d'appel d'une pathologie gastroduodénale. Un questionnaire par patient était rempli par un médecin interne des hôpitaux. Les informations dont on s'enquérait dans le questionnaire comprenaient l’âge, le sexe, la zone de résidence, le statut socio-économique, les antécédents de maladie ulcéreuse et la prise d'anti-inflammatoires non stéroïdiens (AINS). La section du questionnaire concernant la connaissance du statut socio-économique a été conçue pour deux niveaux: élevé et faible. La classe socio-économique moyenne est quasi-inexistante dans notre milieu. Les patients avec un ulcère gastroduodénal compliqué (une hémorragie digestive haute active, une perforation, une sténose) ou ayant reçu un traitement anti sécrétoire et/ou antibiotique lors des quatre dernières semaines précédant l'examen, les patients précédemment traités pour une infection à *H.pylori* ou ayant les antécédents de maladie ulcéreuse et les patients sous un traitement anticoagulant étaient exclus. Le test rapide à l'uréase, kit commercial Pronto Dry^®^ (Medical Instruments Corporation, Solothurn, Switzerland) était la norme diagnostique de l'infection à *H. pylori*. Ce test diagnostique est reconnu pour avoir une sensibilité d'environ 98,1%, une spécificité de 100% et une valeur prédictive positive de 100% [10]. Deux biopsies antrales étaient prélevées et déposées sur le disque du test selon les recommandations du fabriquant. La lecture du test était faite à 5 minutes, 30 minutes et 1 heure après. Le test était positif si le pourtour du disque virait au rouge. L'intensité de la coloration rouge du disque dépendant de la densité de la population de *H.pylori* au site de prélèvement n’était pas une variable d’étude. Les examens de FOGD étaient effectués par trois endoscopistes expérimentés. L'histologie n'a pas été considérée dans cette étude.

A partir des cas décelés, les analyses statistiques ont été effectuées pour évaluer les déterminants de l'infection à *H.pylori* suivants: âge, sexe, statut socio-économique, zone de résidence et prise des AINS. Les symptômes digestifs et les maladies gastroduodénales ont été analysés.

Les données ont été saisies puis analysées à l'aide des logiciels Epi info 6.04 version française et Excel 2007. Pour les variables quantitatives, les moyennes, les écarts types, les médianes et les interquartiles (IQR) ont été calculés. Les proportions ont été établies pour les variables qualitatives avec leurs intervalles de confiance (IC) à 95%.

Pour examiner la relation entre deux variables discrètes, nous avons calculé les risques relatifs (RR) et utilisé le test X^2^ de Pearson avec la correction de Yates et le test exact de Fischer pour les effectifs réduits, pour un seuil de signification admis à 5%.

## Résultats

Au total, 203 patients ont bénéficié d'une FOGD dans la période d’étude. Un total de 171 (84%, 171/203) sujets ont satisfait aux critères d'inclusion et ont été testés pour l'infection à *H.pylori*. Parmi ceux-ci 70 hommes (40,9%) et 101 femmes (59,1%) d’âge moyen 42, 3 ±16,7 ans (étendu 9-82ans), répartis en groupes d’âge de moins de 40 ans 45% (77/171), de 40-50 ans 25% (43/171) et plus de 50 ans 30% (51/171). Par statut socio-économique, 35,1% (60/171) étaient de niveau élevé et 64,9% (111/171) de niveau faible. De la zone de résidence, 22,2% (38/171) vivaient en zone rurale et 77,8% (133/171) vivaient en zone urbaine.

La prévalence globale de l'infection à *H.pylori* était de 72,5% (124/171) (IC à 95% 65,2-79,1). Un total de 55 hommes (78,6%) était positif pour *H.pylori* contre 69 femmes (68,3%) (p = 0,139). La prévalence de l'infection à *H.pylori*variait significativement avec l’âge (p = 0,01). L'infection à *H.pylori* était significativement élevée parmi les sujets de moins de 40 ans avec 83,1% (64/77) comparés au sujets de 40-50 ans 67,4% (29/43) (RR 0,42 IC à 95% 0,16-1,1 p = 0,04 pour les 40-50 ans) et comparés au sujets de plus de 50 ans 60,8% (31/51) (RR 0,73 IC à 95% 0,57-0,93 p = 0,004 pour les plus de 50 ans). La prévalence de l'infection à *H.pylori* était élevée chez les sujets vivant en zone urbaine 75,2% (10/133). Cependant cette augmentation n’était pas statistiquement significative (p = 0,142). De même le statut socio-économique faible était associé à une prévalence légèrement accrue 73,9% (82/111). Cette augmentation n’était pas statistiquement significative (p = 0,588). La prise d'AINS n'influençait pas significativement la prévalence de l'infection à *H.pylori* (p = 0,953 (Tableau 1).

**Tableau 1:** Association between epidemiological risk factors and prevalence of *H. pylori* infection in 171 patients referred for endoscopy (univariate analysis)

Variables and categories	No. of cases (%)	*H. pylori* positive (%)	RR (95%CI)	*p-*value
**Age (years)**				
< 40	77 (45)	64 (83.1)	1	
40-50	43 (25)	29(67.4)	0.42(0.16-1.1)	0.04
>50	51 (30)	31(60.8)	0.73(0.57-0.93)	0.004
**Gender**				
Male	70 (40.9)	55(78.6)	1	
Female	101 (59.1)	69(68.3)	0.87(0.73-1.04)	0.1
**Area of habitation**				
Urban	133 (77.8)	100(75.2)	1	
Rural	38 (22.2)	24(63.2)	0.84(0.65-1.09)	0.1
**Socio-economic status**				
Low	111 (64.9)	82(73.9)	1	
High	60 (35.1)	42(70.0)	0.95(0.78-1.16)	0.6
**NSAIDs user**				
No	117 (68.4)	85(72.6)	1	
Yes	54 (31.6)	39(72.2)	0.99(0.81-1.21)	0.9

Les principales lésions retrouvées parmi les patients étaient la gastrite antrale 49,7% (85/171), la gastrite diffuse 29,8% (51/171), l'UD 15,8% (27/171) et l’œsophagite peptique 11,1% (19/171). Le cancer gastrique était relativement rare parmi les patients 1,2% (2/171). L'examen était normal chez 7,6% de patients (12/171). La prévalence de l'infection à *H.pylori* pour les maladies gastroduodénales était élevée pour les gastrites, l'UD et l’œsophagite peptique. Tous les cas de cancer gastrique étaient *H.pylori* positif (Tableau 2).

**Tableau 2:** Gastro-duodenal pathologies and prevalence of *H. pylori* infection in 171 patients referred for endoscopy.

Endoscopic categories	No. of cases (%)^a^	*H.pylori* positive
		No. of cases (%) a
Normal	12 (7.6)	9 (69.2)
Antrale gastritis	85 (49.7)	62 (72.9)
Corporeal gastritis	7 (4.1)	6 (85.7)
Diffuse gastritis	51 (29.8)	40 (78.4)
Gastric ulcer	8 (4.7)	4 (50.0)
Duodenal ulcer	27 (15.8)	17 (63.0)
GORD	19 (11.1)	12 (63.2)
Hiatal hernia or simple incompetence	15 (8.8)	9 (60.0)
Gastric cancer	2 (1.2)	2 (100)
Others	18 (10.5)	17 (94.4)

a The number of cases respectively exceed 171 and 124 given that some patients had more than one lesion at endoscopy. GORD= gastro-esophageal reflux disease.

L'UD et l'ulcère gastrique (UG) étaient plus fréquents chez les sujets *H.pylori* positif et utilisateurs d'AINS (15,6% et 5,1% respectivement) que chez les sujets *H.pylori* positif et non utilisateurs d'AINS (12,5% et 2,4% respectivement). Cependant l'association entre l'utilisation d'AINS et l'infection à *H.pylori* chez les sujets avec un UD ou un UG n’était pas statistiquement significative (RR 1,19; 0,47-2,98 p = 0,7 pour l'UD et RR 2,18; 0,32-14,9 p = 0,8 pour l'UG).

## Discussion

### *H. pylori*: prévalence et déterminants de l'infection

Le statut socio-économique et les autres déterminants pour l'infection à *H.pylori* ont été analysés dans plusieurs études épidémiologiques, dont la plupart n'ont pas été faites en Afrique subsaharienne [2, 11–13]. Les récentes études confirment l'association entre *H.pylori* et les conditions d'hygiène liées à la pauvreté dans le milieu urbain d'une part, et d'autre part, la prévalence accrue de l'infection à *H.pylori* parmi les individus de race noire vivant en Amérique et ceux issus d'ancêtres africains noirs [14, 15].

Dans notre étude, la prévalence de l'infection à *H.pylori* est particulièrement élevée, surtout chez les sujets de moins de 40 ans et les hommes sans qu'un lien significatif au sexe soit retrouvé. Les déterminants pour l'infection à*H.pylori*, notamment le statut socio-économique et la zone de résidence ne semblent pas influencer significativement l'infection à *H.pylori* parmi les tranches d’âge étudiées ni parmi les sexes. Pour comprendre ces résultats, il faut savoir que les sujets de niveau socio-économique élevé et ceux vivant en zone urbaine sont dans la majorité issus des mêmes niveaux socio-économiques faibles, et que beaucoup vivent indifféremment en ville comme au village. Il s'agit souvent d'une ascension sociale en Afrique pour plusieurs sujets, alors qu'ils ont connus des conditions de vie précaires dans la prime enfance, moment de la contamination. Les résultats de l’étude de Ndip et al. [9], parmi les enfants de 0 à 10 ans en milieu rural au sud-ouest du Cameroun confirment d'une part ce fait d'une infection précoce, mais montrent d'autre part, une différence avec nos résultats sur l'influence du statut socio-économique. La migration des populations rurales vers la ville et leur ascension sociale plus tard expliqueraient nos résultats [16]. Nos résultats sont différents de ceux d'autres auteurs car ils montrent un effet protecteur mais non significatif de la vie en zone rurale. Ceci trouverait l'explication dans les modes de contamination à H.pylori qui comprennent la mauvaise eau et l'insalubrité [2, 17, 18], conditions qui sont souvent réunies dans les villes en Afrique plutôt que dans les villages.

### *H. pylori* et la maladie ulcéreuse

La prévalence de l'infection à *H.pylori* est estimée à plus de 90% pour les UD et 70% pour les UG [3, 19, 20]. Nos résultats indiquent au plus une prévalence de 63% pour les UD et 50% pour les UG. Cette diminution de prévalence des UD liés à *H.pylori* a été rapportée dans plusieurs études en Australie et aux États-Unis d'Amérique [19, 21]. Des facteurs pouvant expliquer une diminution des ulcères liés à *H.pylori* dans notre série, nous pouvons retenir l'absence de biopsies fundiques. Par contre, la consommation d'AINS était certes importante dans notre étude (31,6%), mais cette consommation élevée ne reflète pas la réalité dans la population générale où elle est faible. Aussi les ulcères liés aux AINS ne représentaient que 14,3% contre 17,1% des ulcères non liés ni à *H.pylori* ni aux AINS. Ces derniers ulcères sont en augmentation dans plusieurs études récentes dont celle de Gisbert et al, dans laquelle ils sont qualifiés d'ulcères idiopathiques [21].

### *H. pylori* et le cancer gastrique: l’énigme africaine?

De multiples études épidémiologiques en Asie et en Occident ont confirmé le rôle carcinogène de *H.pylori* [22–24]. Cependant, dans certaines régions comme en Inde ou au Bangladesh il existe un paradoxe entre la prévalence élevée de l'infection à *H.pylori* et une incidence faible pour le cancer gastrique [25]. Cette énigme a longtemps été avancée en Afrique subsaharienne [24, 26–29] et a causé la controverse [30]. La proportion de cancers gastriques distaux attribuables à *H.pylori* a été estimée à 80% dans les pays en voie de développement [3]. Notre étude rapporte une prévalence de 100% de *H.pylori* associé au cancer gastrique. Cependant, parmi les patients *H.pylori* positif seuls 1,6% étaient porteurs de cancer gastrique. Dans la littérature moins de 1% de sujets infectés par *H.pylori* développent un cancer gastrique, qui est un des plus communs et doté d'une mortalité élevée dans le monde. Son incidence est variable selon les régions. Elle est élevée en Chine et en Europe de l'Est, mais faible en Afrique [23, 24, 27]. Au regard d'une prévalence à *H.pylori* élevée et ceci surtout chez les sujets jeunes, on s'attendrait à une prévalence élevée de ce cancer au Cameroun. Ce que nous n'avons pas observé. Ce résultat comme d'autres, participe de l’énigme africaine [24, 26–29].

### *H. pylori* et la pathologie du reflux gastro-œsophagien

La prévalence de l'infection à *H.pylori* a été rapportée moindre chez les sujets souffrant de pathologie du reflux gastro-œsophagien par rapport à une population de témoins [3]. Cependant, aucune corrélation n'a été retrouvée entre l'infection à *H.pylori* et le développement de l’œsophagite peptique ou de modifications de la muqueuse œsophagienne [31]. Par contre, à l'histopathologie, l’œsophage de Barrett serait lié à une prévalence moindre de l'infection à *H.pylori* [31]. Dans notre série, cette prévalence n’était pas différente de celle des sujets avec un examen normal, comme avec celle des sujets souffrant d'un UD. Par contre, la présence de *H.pylori* était associée à un effet protecteur modeste sur l’œsophagite.

### *H. pylori* et prise d'AINS

L'association entre l'infection à *H.pylori* et l'utilisation des AINS dans la maladie ulcéreuse est controversée. *H.pylori* et les AINS sont des facteurs de risque indépendants et synergiques de la maladie ulcéreuse et de l'ulcère hémorragique [32]. Dans une méta-analyse sur le rôle de *H.pylori* et les AINS dans la maladie ulcéreuse, Huang et al. ont également retrouvé cette synergie d'action entre *H.pylori* et les AINS dans le développement de la maladie ulcéreuse et l'ulcère hémorragique [33]. Dans la même étude, les auteurs ont montré que l'ulcère peptique était rare chez les sujets *H.pylori* négatif et non utilisateurs d'AINS. Nos résultats indiquent une prévalence élevée mais non significative chez les patients *H.pylori* positif et utilisateurs d'AINS. L'utilisation d'AINS n’était pas associée à l'infection à *H.pylori* chez les patients souffrant de maladie ulcéreuse. Ce résultat est aussi rapporté dans la méta-analyse récente de Tang et al. [34]. Aussi, nos résultats montrent une prévalence élevée de la maladie ulcéreuse chez les sujets *H.pylori* négatifs et non utilisateurs d'AINS. L'augmentation des ulcères chez les sujets *H.pylori* négatifs et non utilisateurs d'AINS est de plus en plus signalée par d'autres auteurs surtout aux États-Unis [19, 21]

## Conclusion

Notre étude montre une prévalence élevée de l'infection à *H.pylori* au Cameroun. Cette infection est associée au jeune âge et ne dépend pas du genre, du statut socio-économique et de la zone de résidence des individus. Parmi les personnes atteintes, la maladie ulcéreuse, la gastrite et l’œsophagite sont fréquentes. Par contre, le cancer gastrique est rare. Il n'y a pas d'association entre l'infection à *H.pylori* et l'utilisation d'AINS dans la pathogénèse de la maladie ulcéreuse dans notre milieu.
